# Visfatin Increases VEGF-Dependent Angiogenesis of Endothelial Progenitor Cells during Osteoarthritis Progression

**DOI:** 10.3390/cells9051315

**Published:** 2020-05-25

**Authors:** Chun-Hao Tsai, Shan-Chi Liu, Wen-Hui Chung, Shih-Wei Wang, Min-Huan Wu, Chih-Hsin Tang

**Affiliations:** 1 Department of Sports Medicine, College of Health Care, China Medical University, Taichung 404, Taiwan; ritsai8615@gmail.com; 2 Department of Orthopedic Surgery, China Medical University Hospital, Taichung 404, Taiwan; 3 Department of Medical Education and Research, China Medical University Beigang Hospital, Yunlin 651, Taiwan; sdsaw.tw@yahoo.com.tw; 4 Department of Pharmacology, School of Medicine, China Medical University, Taichung 404, Taiwan; sandy780717@gmail.com; 5 Department of Medicine, Mackay Medical College, New Taipei City 252, Taiwan; shihwei@mmc.edu.tw; 6 Graduate Institute of Natural Products, College of Pharmacy, Kaohsiung Medical University, Kaohsiung 807, Taiwan; 7 Physical Education Office, Tunghai University, Taichung 407, Taiwan; 8 Sports Recreation and Health Management Continuing Studies, Tunghai University, Taichung 807, Taiwan; 9 Chinese Medicine Research Center, China Medical University, Taichung 404, Taiwan; 10 Department of Biotechnology, College of Health Science, Asia University, Taichung 41354, Taiwan

**Keywords:** osteoarthritis, visfatin, VEGF, miR-485-5p, angiogenesis

## Abstract

Osteoarthritis (OA) pannus contains a network of neovascularization that is formed and maintained by angiogenesis, which is promoted by vascular endothelial growth factor (VEGF). Bone marrow-derived endothelial progenitor cells (EPCs) are involved in VEGF-induced vessel formation in OA. The adipokine visfatin stimulates the release of inflammatory cytokines during OA progression. In this study, we found significantly higher visfatin and VEGF serum concentrations in patients with OA compared with healthy controls. We describe how visfatin enhanced VEGF expression in human OA synovial fibroblasts (OASFs) and facilitated EPC migration and tube formation. Treatment of OASFs with PI3K and Akt inhibitors or siRNAs attenuated the effects of visfatin on VEGF synthesis and EPC angiogenesis. We also describe how miR-485-5p negatively regulated visfatin-induced promotion of VEGF expression and EPC angiogenesis. In our OA rat model, visfatin shRNA was capable of inhibiting visfatin and rescuing EPC angiogenesis and pathologic changes. We detail how visfatin affected VEGF expression and EPC angiogenesis in OASFs by inhibiting miR-485-5p synthesis through the PI3K and Akt signaling pathways.

## 1. Introduction

Osteoarthritis (OA) is a common age-related and chronic degenerative joint disorder that affects all body joints. Major symptoms of OA include cartilage degradation, osteophyte formation, bone remodeling, neoangiogenesis, and synovial inflammation [1], which are associated with pain, physical disability, and substantial impairments in quality of life. The synovium plays an important role in the pathogenesis of OA. The synthesis of chondrolytic enzymes and proinflammatory mediators by the inflamed synovium leads to cartilage destruction, which in turn enhances synovial inflammation [2,3]. OA synovial fibroblasts (OASFs) sustain arthritic pathology by excreting chondrolytic enzymes and inflammatory mediators [2,4,5]. In theory, synovium-targeted therapy could slow OA progression and mitigate the disease symptoms [6,7].

OA pannus contains a network of neovascularization that is formed and maintained by angiogenesis, which is promoted by vascular endothelial growth factor (VEGF) [8]. New vessel formation also involves endothelial progenitor cells (EPCs) [9,10], which contain the cell surface markers CD133, CD34, and vascular endothelial growth factor receptor-2 (VEGFR2), all of which facilitate postnatal vasculogenesis [11] and exert regenerative effects [12]. VEGF is well recognized for its ability to increase EPC proliferation and migration, besides facilitating angiogenesis [12]. Importantly, VEGF-induced promotion of EPCs facilitates angiogenesis and the development of OA [13,14]. Thus, EPC-dependent angiogenesis shows promise as a new target for OA treatment.

Obesity reportedly increases the risk of developing OA [15,16], although the underlying mechanisms for this risk association are unknown. What is known is that adipokines, multifunctional molecules secreted by the adipose tissue, act as an intersecting link between obesity and OA by modulating the activities of cartilage, synovium, bone, and various immune cells [15,17]. Visfatin is a proinflammatory adipokine produced by visceral white adipose tissue and is found in the bone marrow, skeletal muscles, and liver [18]. Upregulated serum levels of visfatin are found in OA patients [19,20]. Visfatin also plays a role in damage of the synovial joint [16]. The present study aims to investigate the connection between visfatin and EPC-dependent angiogenesis during OA progression. The evidence indicates that visfatin is an appropriate therapeutic target in OA.

## 2. Materials and Methods

### 2.1. Materials

Cell culture supplements were bought from Invitrogen (Carlsbad, CA, USA). Antibodies against p85, Akt, p-p85, p-Akt, VEGF, and β-actin were all bought from Santa Cruz (Santa Cruz, CA, USA). All ON-TARGETplus siRNAs (pool form) were bought from Dharmacon (Lafayette, CO, USA). The miR-485-5p mimic and mimic control were obtained from Thermo Fisher (Waltham, MA, USA). qPCR primers and probes, as well as Taqman^®^ one-step PCR Master Mix, were supplied by Applied Biosystems (Foster City, CA, USA). Ly294002 and the Akt inhibitor (124005) were purchased from Sigma-Aldrich (St. Louis, MO, USA).

### 2.2. Cell Culture

Synovial tissue from the suprapatellar pouch of the knee was obtained from patients diagnosed with Ahlbäck stage IV OA. Synovial fibroblasts were cultured in DMEM medium supplemented with 10% fetal bovine serum (FBS), penicillin (50 units/mL), and streptomycin (50 μg/mL), as previous reports described [21,22].

Human primary EPCs were prepared according to our previously described protocol [23,24]. Cells were maintained in a humidified incubator at 37 °C, 5% CO_2_.

### 2.3. Clinical Samples

Blood samples were obtained from patients with primary OA (we excluded any cases with underlying immuno-inflammatory rheumatic diseases or crystal deposition arthritis) of the knee undergoing knee replacement surgery and also from those undergoing arthroscopy after trauma/mechanical patellofemoral syndrome (who served as healthy controls) in China Medical University Hospital, Taichung, Taiwan. The study protocol was approved by the Institutional Review Board (IRB) of China Medical University Hospital, and all methods were performed in accordance with the IRB’s guidelines and regulations. Informed written consent was obtained from all patients.

### 2.4. RT-qPCR of mRNA and miRNA

Total RNA was extracted from OASFs by TRIzol; reverse transcription used 1 μg of total RNA transcribed into cDNA by oligo (dT) primers. RT-qPCR used the Taqman^®^ One-Step RT-PCR Master Mix [25,26].

### 2.5. Western Blot Analysis

Cell lysates were separated by SDS-PAGE electrophoresis then transferred to polyvinylidene difluoride membranes, following the method described in our previous work [27,28]. After blocking, the membranes were incubated with primary antibodies and then secondary antibodies. Enhanced chemiluminescence of the blots was visualized with the UVP Biospectrum system (UVP, Upland, CA, USA) [29,30,31].

### 2.6. ELISA Assay

OASFs were cultured and stimulated with visfatin for 24 h with or without the transfection of siRNAs or the treatment with inhibitors. The conditioned medium (CM) was collected, and VEGF levels were quantified by a VEGF-A ELISA kit (Peprotech, Rocky Hill, NJ, USA).

### 2.7. Transient Transfection 

Human synovial fibroblasts were cultured in a 6-well plate, and miR-150-5p mimic or visfatinshRNA was transfected into the cells by Lipofectamine™ 2000. 

ON-TARGET*plus* siRNAs (100 nM) was transiently transfected with DharmaFECT1 transfection reagent, according to the manufacturer’s instructions. 

### 2.8. Plasmid Construction and Luciferase Assays

Wild-type and mutant VEGF 3′-UTR plasmids were obtained from Invitrogen (Carlsbad, CA, USA). Luciferase activity was examined using the method described in our previous reports [2,21,32].

### 2.9. EPC Migration and Tube Formation Assays

EPCs were treated with OASF CM for 24 h. EPC migration and tube formation were examined using the methods described in our previous study [33].

### 2.10. In Vivo Matrigel Plug Assay

Four-week-old male nude mice were subcutaneously injected with 0.15 mL of Matrigel containing the indicated OASF CM. On day 7, the Matrigel plugs were harvested, and hemoglobin concentrations were measured according to previously described procedures [14,34,35].

### 2.11. Experimental OA Model

Sprague–Dawley (SD) rats (8 weeks of age, weighing 300–350 g) were procured from the National Laboratory Animal Center in Taiwan and maintained under conditions complying with the Guidelines of the Animal Care Committee of China Medical University, Taichung, Taiwan. We followed an established protocol for our anterior cruciate ligament transection (ACLT) rat model to induce OA [36]. In brief, the left knee was prepared in a surgically sterile fashion. The ACL fibers were transected with a scalpel, and the entire medial meniscus was excised via the medial parapatellar mini-arthrotomy approach. The joint surface was washed with sterile saline solution, and both capsule and skin were sutured after ACL transection and medial meniscectomy. The left knee joint served as the sham-operated control. After surgery (day 0), the rats were divided into 3 groups: a control group, a control shRNA-transfected ACLT group, and a visfatin shRNA-transfected ACLT group. For 6 weeks, the control shRNA-transfected ACLT group and visfatin shRNA-transfected ACLT group were given weekly intra-articular injections of ~7.1 × 10^6^ plaque-forming units (PFU) of control and visfatin shRNA. All rats were allowed to move freely in plastic cages until necropsy at 10 weeks post-surgery.

### 2.12. Micro-Computed Tomography (Micro-CT) Imaging

The micro-computed tomography (micro-CT) assessment protocol was based upon our previous publications [14,35]. Rat knee joints were extracted promptly after sacrifice and fixed in 3.7% formaldehyde for micro-CT imaging. Three-dimensional microstructural volumes from micro-CT scans were analyzed by Skyscan software (CTAn; Bruker) [14].

### 2.13. Statistics

All statistical analyses were carried out using GraphPad Prism 5.0 (GraphPad Software), and all values are expressed as mean ± S.D. Differences between selected pairs from the experimental groups were analyzed for statistical significance using the paired sample *t*-test for in vitro analyses and one-way ANOVA followed by Bonferroni testing for in vivo analyses. Correlations between plasma VEGF and visfatin were calculated using Spearman’s rank correlation coefficient (R). The statistical difference was considered to be significant if the *p*-value was <0.05.

## 3. Results

### 3.1. A Positive Correlation Exists between Visfatin and VEGF Expression in OA

**Figure 1:**
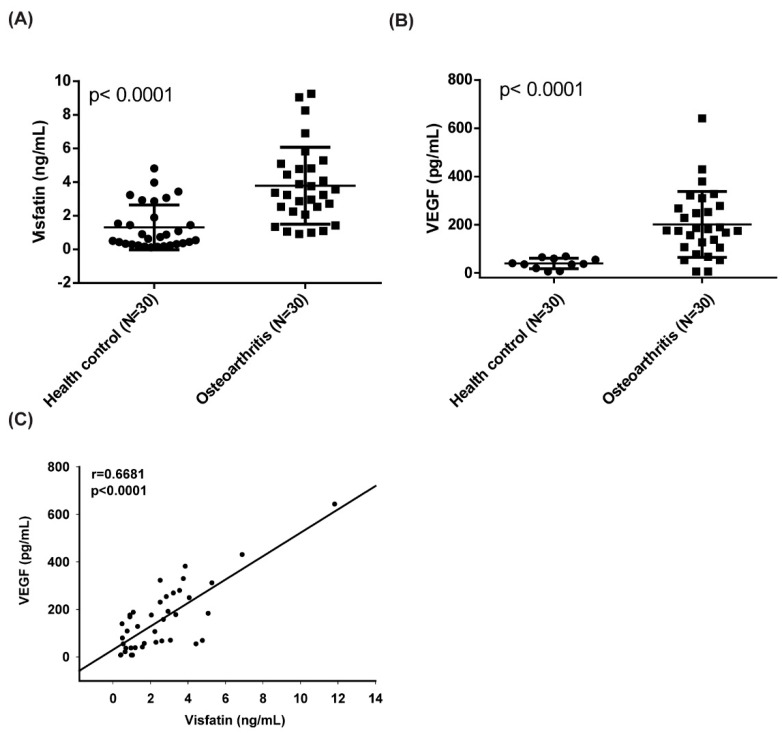
Visfatin expression is positively correlated with vascular endothelial growth factor (VEGF) expression in OA patients. (**A**,**B**) ELISA analysis showing higher serum visfatin and VEGF levels in osteoarthritis (OA) patients (*n* = 30) compared with healthy controls (*n* = 30). Mann–Whitney testing was applied in Figure 1A,B. (**C**) Correlation between levels of visfatin and VEGF expression in serum samples retrieved from OA patients.

In our initial exploration of visfatin and VEGF expression in OA development, ELISA test results revealed significantly higher visfatin and VEGF serum concentrations in patients with OA compared with healthy controls (Figure 1A,B and Supplementary Material Tables S1 and S2). Serum visfatin and VEGF concentrations were positively correlated (Figure 1C).

### 3.2. Visfatin Increases VEGF Expression and EPC Angiogenesis in Human OASFs

**Figure 2:**
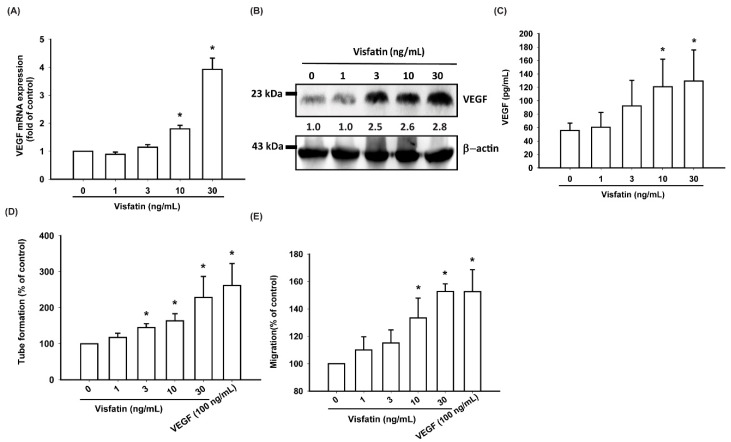
Visfatin stimulates VEGF expression and endothelial progenitor cells (EPC) angiogenesis in OA synovial fibroblasts (OASFs). (**A**–**C**) OASFs were incubated with visfatin (1–30 ng/mL) for 24 h, and VEGF expression was examined by RT-qPCR, Western blot, and ELISA analysis. (**D**,**E**) The conditioned medium (CM) was then collected and applied to EPCs. EPC tube formation and migration were measured; * *p* < 0.05 compared with the control group.

No detailed information exists regarding any crosstalk between visfatin and VEGF in the pathogenesis of OA or on how such an interaction may influence EPC angiogenesis. Here, we found that visfatin (1–30 ng/mL) dose-dependently stimulated transcription of VEGF mRNA and VEGF translation at the protein level (Figure 2A,B) as well as the excretion of the VEGF protein by OASFs (Figure 2C).

As the formation of new blood vessels depends on the migration of EPCs through the capillary basement membrane [37], we analyzed the role of visfatin in EPC migratory activity. The Transwell assay revealed a dramatic increase in EPC migration after their incubation with CM from visfatin-treated OASFs, while the tube formation assay showed that visfatin-treated OASFs dose-dependently facilitated the formation and reorganization of capillary-like network structures (Figure 2D,E; Supplementary Material Figure S1).

### 3.3. Visfatin Promotes VEGF Production and EPC Angiogenesis via the PI3K and Akt Signaling Pathways

**Figure 3:**
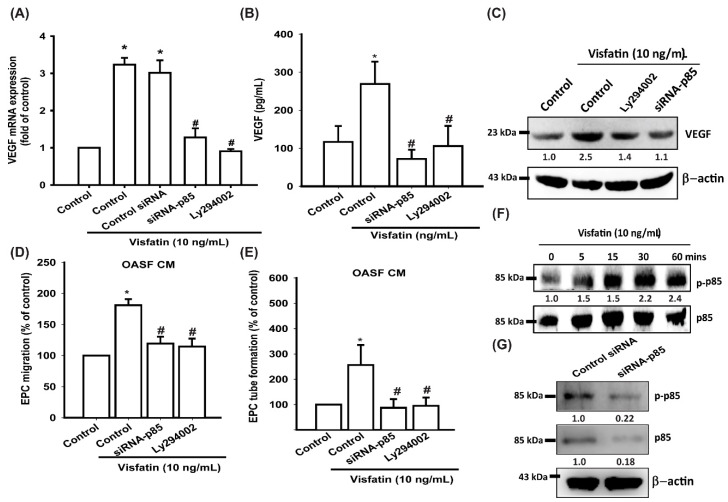
The PI3K pathway is involved in visfatin-induced VEGF synthesis and EPC angiogenesis. (**A**–**C**) OASFs were pretreated with a PI3K inhibitor (Ly294002) or transfected with p85 siRNA, then incubated with visfatin for 24 h. VEGF levels were examined by RT-qPCR, ELISA, and Western blot. (**D**,**E**) The CM was then collected and applied to EPCs. EPC tube formation and migration were measured. (**F**) OASFs were incubated with visfatin for the indicated time intervals, and p85 phosphorylation was examined by Western blot. (**G**) OASFs were transfected with p85 siRNA, and p85 and p-p85 expression was examined by Western blot; * *p* < 0.05 compared with the control group; ^#^ *p* < 0.05 compared with the visfatin-treated group.

The PI3K signaling pathway modulates several cellular functions, including angiogenesis [33,38]. We explored the role of PI3K in visfatin-enhanced VEGF expression by pretreating OASFs with a PI3K inhibitor (Ly294002) or transfecting them with p85 siRNA. Quantitative reverse transcription PCR (RT-qPCR), ELISA, and Western blot assays confirmed that the PI3K inhibitor and p85 siRNA not only significantly reduced visfatin-increased VEGF expression in OASFs (Figure 3A–C) but also inhibited visfatin-promoted EPC migration and tube formation (Figure 3D,E). Western blot analysis demonstrated that visfatin time-dependently promoted p85 phosphorylation (Figure 3F). Transfection of cells with p85 siRNA reduced p85 expression and phosphorylation (Figure 3G).

**Figure 4:**
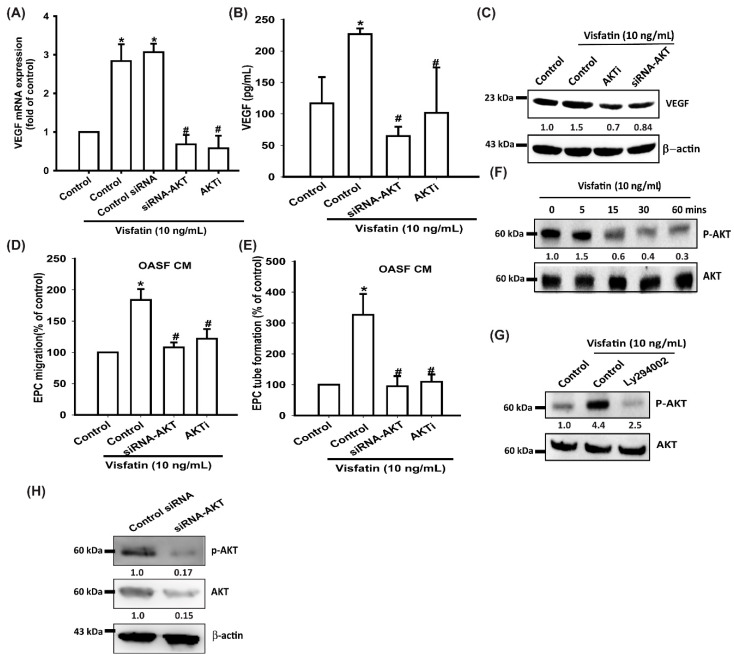
The Akt pathway is involved in visfatin-induced VEGF synthesis and EPC angiogenesis. (**A**–**C**) OASFs were pretreated with an Akt inhibitor or transfected with Akt siRNA, then incubated with visfatin for 24 h. VEGF levels were examined by RT-qPCR, ELISA and Western blot. (**D**,**E**) The CM was then collected and applied to EPCs. EPC tube formation and migration were measured. (**F**,**G**) OASFs were incubated with visfatin for the indicated time intervals or pretreated with a PI3K inhibitor and then stimulated with visfatin, and Akt phosphorylation was examined by Western blot. (**H**) OASFs were transfected with Akt siRNA, then Akt and p-Akt expression was examined by Western blot; * *p* < 0.05 compared with the control group; ^#^ *p* < 0.05 compared with the visfatin-treated group.

Akt is a common downstream signaling molecule of PI3K and a mediator of EPC angiogenesis [39,40]. When we treated OASFs with an Akt inhibitor or transfected them with Akt siRNA prior to visfatin administration, we observed marked reductions in visfatin-induced increases in VEGF expression, EPC migration, and tube formation (Figure 4A–E). In Western blot analysis, visfatin time-dependently promoted Akt phosphorylation (Figure 4F), which was inhibited by the treatment with a PI3K inhibitor (Figure 4G). Transfection of cells with Akt siRNA reduced Akt expression and phosphorylation (Figure 4H).

### 3.4. Visfatin Increases VEGF Production and EPC Angiogenesis via the Inhibition of miR-485-5p Synthesis

**Figure 5:**
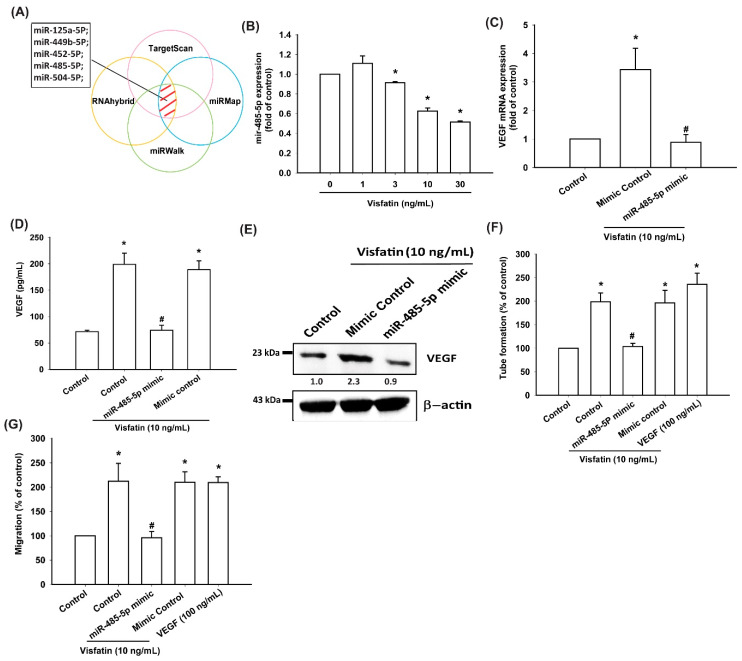
Visfatin promotes VEGF production and EPC angiogenesis by suppressing miR-485-5p. (**A**) Open-source software (TargetScan, miRMap, RNAhybrid, and miRWalk) were used to identify which miRNAs could possibly interfere with VEGF transcription. (**B**) OASFs were incubated with visfatin (1–30 ng/mL). miR-485-5p expression was examined by RT-qPCR. (**C**–**E**) OASFs were transfected with the miR-485-5p mimic and then stimulated with visfatin. VEGF levels were examined by RT-qPCR, ELISA, and Western blot. (**F**,**G**) The CM was then collected and applied to EPCs. EPC tube formation and migration was measured; * *p* < 0.05 compared with the control group; ^#^ *p* < 0.05 compared with the visfatin-treated group.

miRNA expression patterns differ between OA and normal cartilage, and several miRNAs are implicated in OA pathogenesis [22,41]. Using open-source software (TargetScan, miRMap, RNAhybrid, and miRWalk), we sought to identify miRNAs that could potentially interfere with VEGF transcription. Of five candidate miRNAs that we found could bind to the 3′untranslated region (UTR) of VEGF mRNA, miR-485-5p expression was reduced to the greatest extent after visfatin administration (Figure 5A; Supplementary Material Figure S2). Stimulating OASFs with visfatin concentration-dependently inhibited miR-485-5p expression (Figure 5B). To further determine whether visfatin stimulates VEGF expression and EPC angiogenesis by inhibiting miR-485-5p expression, we transfected OASFs with an miR-485-5p mimic and observed reductions in visfatin-enhanced VEGF expression, EPC migration, and tube formation (Figure 5C–G).

**Figure 6:**
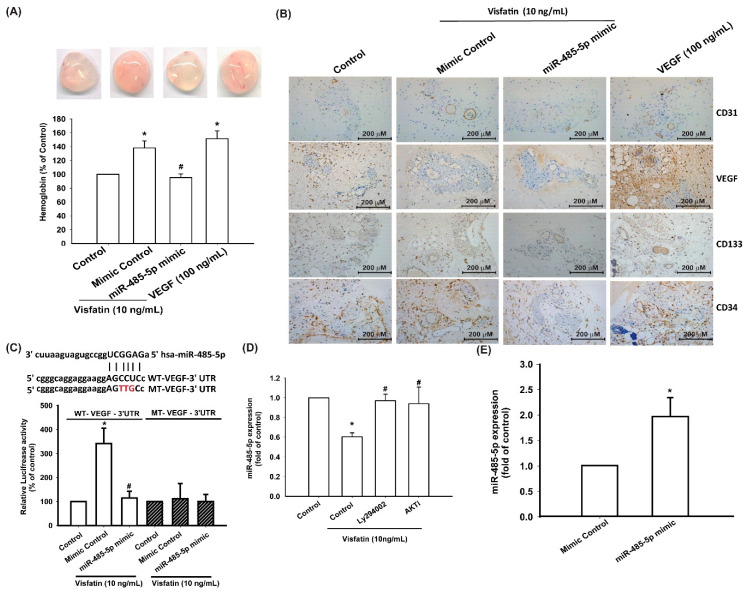
Visfatin suppresses miR-485-5p synthesis via the PI3K and Akt pathways. (**A**) Matrigel plugs containing OASF CM were subcutaneously injected into the flanks of nude mice. After 7 days, the plugs were photographed, and hemoglobin levels were quantified. (**B**) Specimens from the plugs were immunostained with antibodies against CD31, VEGF, CD34, and CD133. (**C**) Schematic 3′UTR representation of human VEGF containing the miR-485-5p binding site. (**D**) OASFs were transfected with the indicated luciferase plasmid with or without the miR-485-5p mimic, then stimulated with visfatin. Relative luciferase activity was examined. (**E**) OASFs were transfected with the miR-485-5p mimic, and miR-485-5p expression was examined by qPCR; * *p* < 0.05 compared with the control group; ^#^ *p* < 0.05 compared with the visfatin-treated group.

We then sought to determine whether visfatin promotes EPC angiogenesis by inhibiting miR-485-5p in vivo. The Matrigel plug assay showed that CM from visfatin-treated OASFs increased vessel formation in vivo (Figure 6A), while the miR-485-5p mimic abolished visfatin-induced promotion of vessel formation (Figure 6A). Immunohistochemistry (IHC) staining indicated that the miR-485-5p mimic antagonized visfatin-facilitated increases in the expression of the vessel marker CD31 and of the levels of VEGF and the EPC-specific markers CD34 and CD133 (Figure 6B).

To determine whether miR-485-3p controls transcription of the *VEGF* gene, we examined the effects of a luciferase reporter vector containing the wild-type 3′UTR of VEGF mRNA (wt-VEGF-3′UTR) and a mutated vector harboring mismatches in the predicted miR-485-3p binding site (mt-VEGF-3′UTR) (Figure 6C). The miR-485-5p mimic inhibited visfatin-increased luciferase activity in the wt-VEGF-3′UTR plasmid but not in the mt-VEGF-3′UTR plasmid (Figure 6C), indicating that miR-485-5p suppresses VEGF transcription through binding to the 3’UTR region of human VEGF mRNA. In addition, the PI3K and Akt inhibitors markedly reversed visfatin-reduced miR-485-5p expression (Figure 6D). Transfection of cells with the miR-485-5p mimic increased miR-485-5p expression (Figure 6E).

### 3.5. Visfatin Knockdown Mitigates EPC Angiogenesis and OA Severity In Vivo

**Figure 7:**
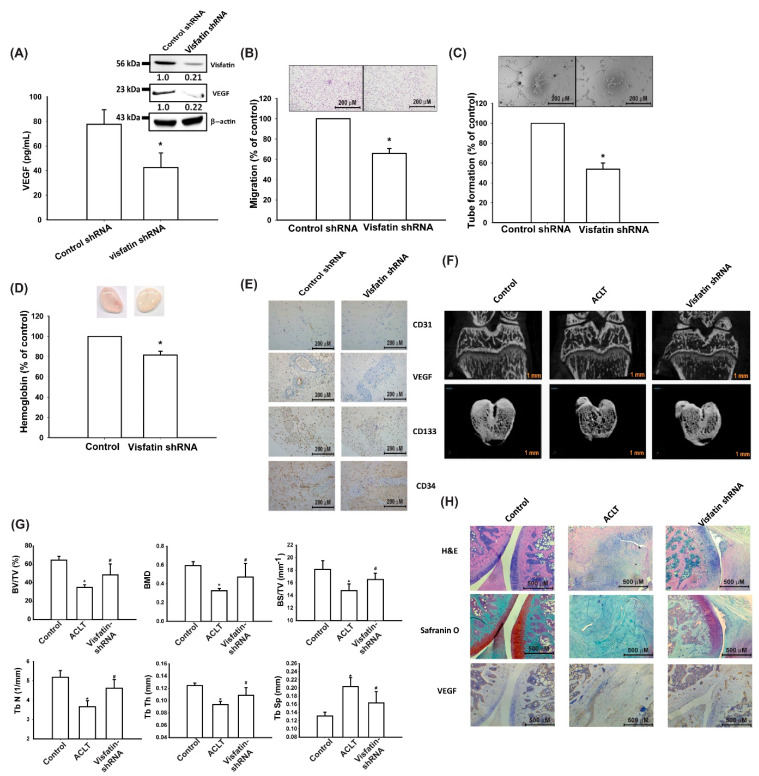
Visfatin knockdown reduces EPC angiogenesis and pathological severity of OA. (**A**) OASFs were transfected with visfatin shRNA. Visfatin and VEGF expression was examined by Western blot and ELISA. (**B**,**C**) The CM was then collected and applied to EPCs. EPC tube formation and migration were measured. (**D**) Matrigel plugs containing OASF CM were subcutaneously injected into the flanks of nude mice. After 7 days, the plugs were photographed, and hemoglobin levels were quantified. (**E**) Specimens from the plugs were immunostained with antibodies against CD31, VEGF, CD34, and CD133. (**F**) Micro-CT images of the control knee, ACLT knee, and visfatin shRNA-transfected ACLT knee. (**G**) Micro-computed tomography (CT) parameters, including bone volume (BV), bone mineral density (BMD), bone surface (BS), trabecular (Tb) number and thickness (Th), and space. (**H**) Specimens from the knee were immunostained with Safranin-O, and anti-VEGF antibodies; * *p* < 0.05 compared with the control group; ^#^ *p* < 0.05 compared with the visfatin-treated group.

The transfection of human OASFs with visfatin shRNA reduced visfatin and VEGF expression (Figure 7A), EPC migration and tube formation (Figure 7B,C), and angiogenesis in vivo (Figure 7D,E).

We then investigated the effects of shRNA-mediated visfatin knockdown on OA severity in our ACLT model. Micro-CT imaging revealed that visfatin shRNA restored the integrity of subchondral bone architecture in ACLT rats (Figure 7F). In comparison to the control samples, subchondral bone from rats with ACLT-induced OA that underwent control shRNA transfection had significantly lower bone volume (BV), BMD (bone mineral density), bone surface (BS), and trabecular (Tb) number and thickness (Th) and higher Tb spacing (Figure 7G). All of these ACLT-induced effects were reversed by visfatin shRNA transfection. IHC and Safranin-O staining demonstrated lower cartilage thickness and significantly higher expression of VEGF in ACLT samples. ACLT-induced histologic changes were reversed by visfatin shRNA transfection (Figure 7H).

## 4. Discussion

OA is the most common form of arthritis and a primary cause of disability [1]. Although much is unclear as to the pathogenesis of OA, it is known that synovium inflammation plays a pivotal role [42], so synovium-targeted therapy could theoretically slow OA progression and lessen the severity of symptoms [6,43]. Pannus formation and neovascularization also play important roles in OA development [8]. VEGF-induced stimulation of angiogenesis is a critical step during OA progression [8,44]. We have previously reported that visfatin promotes the production of the proinflammatory cytokines IL-6 and TNF-α in OASFs [16]. However, the effects of visfatin on VEGF expression and EPC angiogenesis are not clear. In this study, we found higher levels of visfatin and VEGF in patients with OA compared with healthy controls. We also found that visfatin stimulates EPC angiogenesis in OASFs by increasing VEGF expression after inhibiting miR-485-5p synthesis through the PI3K and Akt signaling pathways.

Previous research has demonstrated the upregulated visfatin concentrations in synovial fluid from OA patients compared with that from healthy individuals [19,20]. In this study, we confirmed that visfatin and VEGF levels are higher in serum from OA patients than in that from healthy controls. Our investigation revealed positive correlations between visfatin and VEGF concentrations. According to the evidence presented, VEGF may serve as a target molecule for the visfatin signaling pathway, which facilitates EPC angiogenesis in human OASFs. Knockdown of visfatin inhibited ACLT-induced OA and the expression of EPC and vessel markers in vivo. Visfatin is clearly a critical molecular target in OA therapy. To the best of our knowledge, no investigations have identified any specific visfatin receptor. Thus, further explorations are needed to determine which receptors mediate visfatin-induced VEGF expression and EPC angiogenesis.

The activation of the PI3K signaling pathway is essential for regulating multiple cellular functions [45], including angiogenesis and metastasis [33,46]. Our evidence shows that visfatin facilitates p85 phosphorylation, while a PI3K inhibitor and siRNA treatment diminished visfatin-enhanced VEGF production. These compounds effectively attenuated visfatin-induced promotion of EPC migration and tube formation. PI3K-dependent Akt activation is critical for controlling angiogenesis [47,48]. We found that an Akt inhibitor and siRNA treatment reversed visfatin-induced increases in VEGF expression and EPC migration and tube formation, indicating that Akt is required for visfatin-promoted VEGF production and EPC angiogenesis. Our findings also reveal that visfatin facilitates Akt phosphorylation. PI3K inhibitor treatment reduced visfatin-induced Akt phosphorylation, suggesting that PI3K-dependent Akt activation regulates visfatin-mediated VEGF expression and EPC angiogenesis in human OASFs. However, we did not examine which upstream molecules of PI3K were affected by visfatin stimulation. How visfatin activates PI3K/Akt signaling requires further examination.

It has been stated that miRNAs effectively post-transcriptionally regulate gene expression [49]. In OA, several miRNAs show aberrant expression levels and are capable of regulating the expression of inflammatory pathways [49]. It is speculated that pharmacotherapy capable of regulating miRNA expression would reduce the inflammatory process in OA and assist with the management of this disease [49,50]. We searched open-source miRNA software to determine whether miR-485-5p interferes with VEGF transcription. We found that visfatin stimulation reduces miR-485-5p synthesis and that transfecting OASFs with an miR-485-5p mimic mitigates visfatin-stimulated VEGF expression and EPC angiogenesis. PI3K and Akt inhibitor treatments rescued visfatin-induced inhibition of miR-485-5p expression, indicating that visfatin facilitates VEGF production and EPC angiogenesis by reducing miR-485-5p expression via the PI3K and Akt signaling cascades.

Histopathological features of OA that are illustrated by the ACLT model include articular cartilage destruction and infiltration of inflammatory cells [36]. In this study, micro-CT imaging and IHC staining of the ACLT joints revealed articular cartilage erosion, which was attenuated by visfatin shRNA. IHC staining also revealed that visfatin shRNA reduced the levels of a vessel marker (VEGF), suggesting that the effect of visfatin in ACLT rats might be due to the expression of angiogenic mediators.

In order to ensure the confidentiality and anonymity of our study participants, we did not record demographic details of age, gender, body mass index, or any other general information. Lacking these details means that we were unable to compare demographic data with visfatin and VEGF expression.

## 5. Conclusions

Our study shows that visfatin increases VEGF expression and promotes EPC angiogenesis in OASFs by inhibiting miR-485-5p synthesis via PI3K and Akt signaling. Thus, visfatin is an appropriate therapeutic target in OA. Our results enrich our knowledge about the involvement of OASFs in OA and may lead to more effective therapies. Further studies are needed to explore which antibodies or small molecules against visfatin could be used to treat OA.

## Supplementary Materials

The following are available online at https://www.mdpi.com/2073-4409/9/5/1315/s1, Figure S1: The image of figure 2D and 2E, Figure S2. The miRNAs expression after Visfatin treatment in human OASFs, Table S1: Visfatin levels in serum (Mann-Whitney test), Table S2: VEGF levels in serum (Mann-Whitney test).
