# Supplementary material for: Visfatin Increases VEGF-Dependent Angiogenesis of Endothelial Progenitor Cells during Osteoarthritis Progression
Source: Cells. 2020 May 25;9(5):1315. doi: 10.3390/cells9051315 (PMC7291153; doi:10.3390/cells9051315)
Supplement: Supplementary file 1 [file cells-09-01315-s001.pdf]

**Supplementary Data**

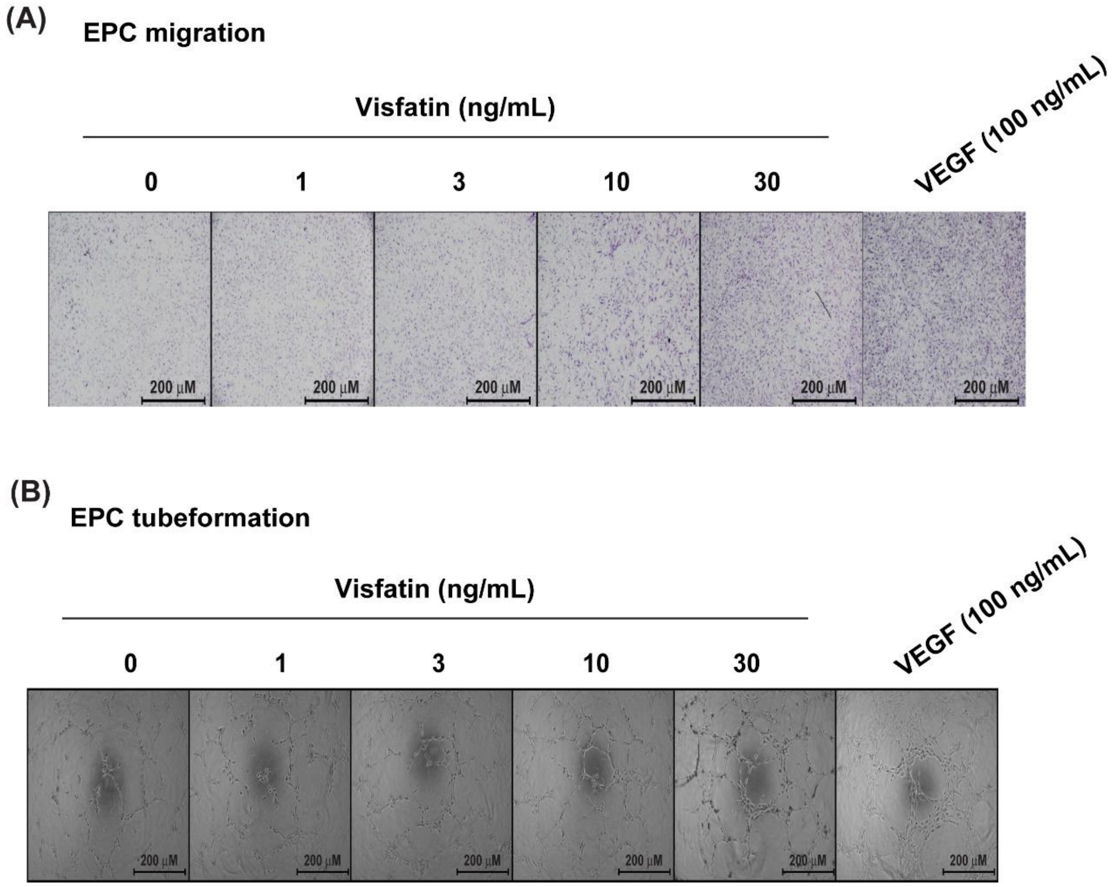


**Fig. S1. The image of figure 2D and 2E.**

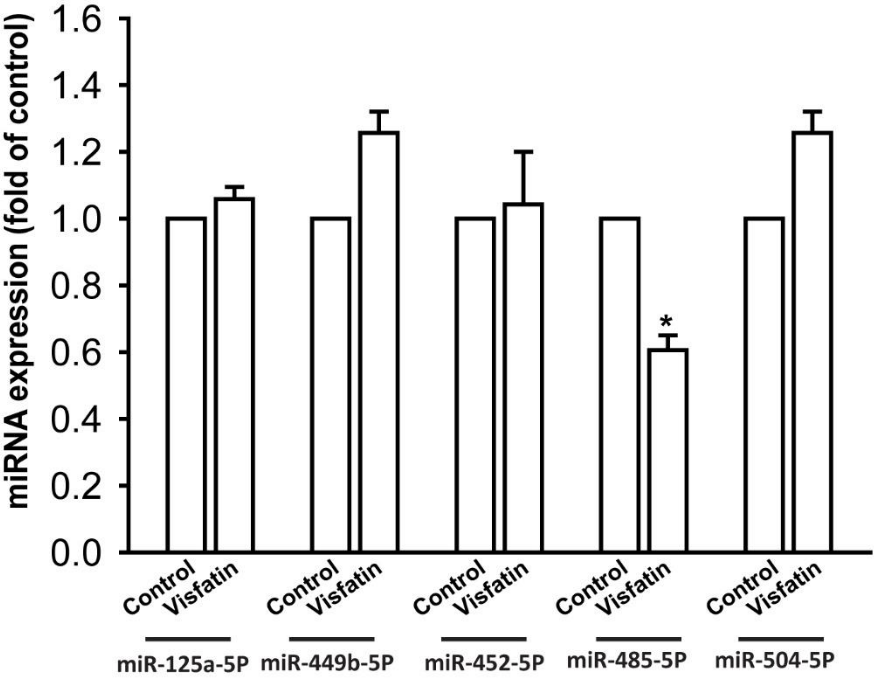


**Fig. S2. The miRNAs expression after Visfatin treatment in human OASFs.** The OASFs were incubated with visfatin (10 ng/mL) for 24 h, and miRNAs expression were examined by qPCR. Results are expressed as the mean ± S.D. *, $p < 0.05$ compared with control.

**TABLE 1. VISFATIN LEVELS IN SERUM (MANN-WHITNEY TEST)**

| SAMPLE | Health control | Osteoarthritis |
|---|---|---|
| **N** | 30 | 30 |
| **MINIMUM** | 0.1159 | 0.9187 |
| **MEDIAN** | 0.6802 | 3.302 |
| **MAXIMUM** | 4.815 | 9.257 |
| **IQR** | 1.8216 | 2.681 |
| **P VALUE** | 0.0001 | 0.0155 |

**TABLE 2. VEGF LEVELS IN SERUM (MANN-WHITNEY TEST)**

| SAMPLE | Health control | Osteoarthritis |
|---|---|---|
| **N** | 30 | 30 |
| **MINIMUM** | 5.848 | 5.848 |
| **MEDIAN** | 36.74 | 176 |
| **MAXIMUM** | 68.2 | 641.4 |
| **IQR** | 40.25 | 166.7 |
| **P VALUE** | 0.3842 | 0.0357 |
